# Diagnostic Characteristics of Lactate Dehydrogenase on a Multiplex Assay for Malaria Detection Including the Zoonotic Parasite *Plasmodium knowlesi*

**DOI:** 10.4269/ajtmh.21-0532

**Published:** 2021-11-15

**Authors:** Rebecca Barney, Miguel Velasco, Caitlin A. Cooper, Andrew Rashid, Dennis E. Kyle, Robert W. Moon, Gonzalo J. Domingo, Ihn Kyung Jang

**Affiliations:** ^1^ Diagnostics, PATH, Seattle, Washington;; ^2^ Department of Infectious Diseases and Center for Tropical and Emerging Global Diseases, University of Georgia, Athens, Georgia;; ^3^ Faculty of Infectious and Tropical Diseases, London School of Hygiene and Tropical Medicine, London, United Kingdom

## Abstract

*Plasmodium* lactate dehydrogenase (pLDH) is a common target in malaria rapid diagnostic tests (RDTs). These commercial antibody capture assays target either *Plasmodium falciparum*–specific pLDH (*Pf*LDH), *P. vivax*–specific pLDH (*Pv*LDH), or a conserved epitope in all human malaria pLDH (PanLDH). However, there are no assays specifically targeting *P. ovale, P. malariae* or zoonotic parasites such as *P. knowlesi* and *P. cynomolgi*. A malaria multiplex array, carrying the specific antibody spots for *PfLDH, PvLDH,* and PanLDH has been previously developed. This study aimed to assess potential cross-reactivity between pLDH from various *Plasmodium* species and this array. We tested recombinant pLDH proteins, clinical samples for *P. vivax, P. falciparum, P. ovale curtisi,* and *P. malariae;* and in vitro cultured *P. knowlesi* and *P. cynomolgi*. *P. ovale*-specific pLDH (*Po*LDH) and *P. malariae*-specific pLDH (*Pm*LDH) cross-reacted with the *Pf*LDH and PanLDH spots. *Plasmodium Knowlesi*-specific pLDH (*Pk*LDH) and *P. cynomolgi*-specific pLDH (*Pc*LDH) cross-reacted with the *Pv*LDH spot, but only *Pk*LDH was recognized by the PanLDH spot. *Plasmodium ovale* and *P. malariae* can be differentiated from *P. falciparum* by the concentration ratios of PanLDH/*Pf*LDH, which had mean (range) values of 4.56 (4.07–5.16) and 4.56 (3.43–6.54), respectively, whereas *P. falciparum* had a lower ratio of 1.12 (0.56–2.61). *Plasmodium knowlesi* had a similar PanLDH/*Pv*LDH ratio value, with *P. vivax* having a mean value of 2.24 (1.37–2.79). The cross-reactivity pattern of pLDH can be a useful predictor to differentiate certain *Plasmodium* species. Cross-reactivity of the pLDH bands in RDTs requires further investigation.

## INTRODUCTION

Global malaria programs have been focused largely on *Plasmodium falciparum* in sub-Saharan Africa and to a lesser extent *P. vivax* in Asia and South America.^1^ However, the success of malaria elimination requires stopping transmission of all human-infecting *Plasmodium* species—such as *P. falciparum*, *P. vivax*, *P. malariae*, and *P. ovale* spp. (*P. ovale curtisi* of classic type and *P. ovale wallikeri* of variant type)—that cause natural human malaria via parasite speciation and treatment of infection.^2^^–^^4^ *P. malariae* and *P. ovale* spp. are prevalent in Africa and other malaria-endemic regions but receive little attention because of clinically mild symptoms.^5^^,^^6^

In addition, malaria can be caused in humans by simian parasites such as *P. knowlesi* and *P. cynomolgi*. *P. knowlesi*, commonly found in natural hosts including long-tailed macaques (*Macaca fascicularis*) and pig-tailed macaques (*M. nemestrina*) in Southeast Asian countries, has been documented in humans as a result of increased human–macaque contacts as a result of habitat disruption and destruction by extensive population growth and deforestation in the areas where natural hosts and the anopheline mosquito vectors carrying *P. knowlesi* exist.^7^^,^^8^ *P. cynomolgi*, often detected with other simian parasites such as *P. inui*, *P. coatneyi*, or *P. fieldi* in the macaque population in Southeast Asia, is a close relative of *P. vivax*.^9^^,^^10^ Natural human infection with *P. cynomolgi* has been reported in Southeast Asia.^11^^–^^13^

Severe malarial symptoms and even death have been described with human *P. knowlesi* infection.^14^^–^^16^ *P. knowlesi* has a relatively short asexual life cycle of approximately 24 hours, compared with approximately 48 hours for *P. falciparum*, *P. vivax*, and *P. ovale*, and 72 hours for *P. malariae*.^17^ Consequently, *P. knowlesi* infection can rapidly replicate to reach a high parasite density and progress to severe malaria similar to that from *P. falciparum* if there is no prompt medical treatment.^18^^,^^19^ Although cases of *P. knowlesi* (misdiagnosed as *P. malariae*) may have been prevalent at low levels for many decades, a relative surge has arisen as a result of a dramatic reduction in prevalence of *P. vivax* and *P. falciparum* over the past decade.^20^^,^^21^ Identification of *P. knowlesi* by microscopy is unreliable largely because of its similar morphological features with *P. falciparum* at the early trophozoite stage and to *P. malariae* at the other stages, notably trophozoite band forms.^22^^,^^23^

In addition to the limitations of microscopy to accurately identify *P. knowlesi*, there are no rapid diagnostic tests (RDTs) specifically designed for *P. knowlesi* identification. Currently available malaria RDTs use several key biomarkers to differentiate between species: *P. falciparum-*specific histidine-rich protein 2 (HRP2) or/and lactate dehydrogenase (*Pf*LDH) for *P. falciparum,* and *P. vivax*-specific lactate dehydrogenase (*Pv*LDH) for *P. vivax*. Some malaria RDTs detect pan-malarial biomarkers such as the pan-*Plasmodium* lactate dehydrogenase (pLDH) or *Plasmodium* aldolase (pAldo) that can, in principle, detect but not differentiate all *Plasmodium* species.^24^ Several studies demonstrated a risk of cross-reactivity in commercial malaria RDTs (OptiMAL-IT^®^ [Bio-Rad, Hercules, CA], Entebe Malaria Cassette, BinaxNOW^TM^ Malaria [Abbott, Abbott Park, IL], and Paramax-3^TM^ [Tulip Diagnostics Ltd, Goa, India]) between parasites of two major *Plasmodium* species and *P. knowlesi* parasites, and poor sensitivity in cases of low parasitemia with *P. knowlesi* infection, revealing the need for caution in data interpretation if confirmatory testing is not conducted.^25^^,^^26^ For more accurate identification of *P. knowlesi,* several groups reported the stronger performance of molecular techniques, such as nested polymerase chain reaction (PCR) assay, loop-mediated isothermal amplification (LAMP), and real-time PCR assays.^27^^–^^30^ However, these molecular tests are not simple to use or cost-effective. Similarly, there are no *P. ovale-* and *P. malariae*-specific RDTs, also leading to the underreporting of these more widely spread forms of human malaria.^31^^,^^32^ Interestingly, an immunocapture assay has been used to evaluate specificity of monoclonal antibodies against the pLDH isoforms from *Plasmodium* species.^33^^,^^34^ A similar reactivity pattern with the pLDH proteins of *P. cynomolgi* (*Pc*LDH) and *P. vivax* (*Pv*LDH) indicated the close similarity of pLDH isoforms, whereas the absence of reactivity of the *P. falciparum*-specific antibodies with other simian parasites except *P. knowlesi* suggested the possible use of these antibodies to distinguish *P. knowlesi* from the other simian parasites.

The pLDH is the primary target analyte for *P. vivax* diagnosis with RDTs.^35^ With the emergence of *hrp2/hrp3* gene deletions in *P. falciparum* infections leading to false negative RDT results, pLDH is increasingly a key biomarker for RDT-based diagnosis of malaria.^36^^,^^37^ It is important to understand the reactivity of different *Plasmodium* species to the different pLDH assays used in RDTs—namely *P. falciparum* pLDH, *P. vivax* pLDH, and PanLDH. In previous studies, we described the development and validation of a laboratory-based multiplex platform, the Human Malaria Array (5-Plex; Quansys Biosciences, Logan, UT), that can quantify HRP2 and pLDH from *P. falciparum* and *P. vivax*.^38^^,^^39^ This multiplex immunoassay, if applied to characterize clinical as well as in vitro cultured samples from these closely related but less-common *Plasmodium* species, may increase our understanding of the performance of current RDTs in these species.

Here, analysis of cross-reactivity of this reference assay was investigated with clinical samples of *P. malariae* and *P. ovale* and by using culture-adapted strains of both *P. cynomolgi* and *P. knowlesi*.^40^^,^^41^ We also explored the indicators that can differentiate these less-common parasites from *P. falciparum* and *P. vivax* parasites, and the time-course expression of *P. knowlesi*-specific pLDH (*Pk*LDH) protein in culture by a multiplex reference assay.

## MATERIALS AND METHODS

### Clinical samples.

A total of 116 clinical blood samples (29 *P. falciparum*, 62 *P. vivax*, 17 *P. malariae*, and 8 *P. ovale*) from febrile patients with malaria mono-infection were obtained from the Foundation for Innovative New Diagnostics (FIND) Specimen Bank. Nested PCR with microscopy was used to identify the species of human malaria parasites in the samples. Parasite density in clinical samples was confirmed by either microscopy or quantitative PCR (qPCR) methods.

### In vitro *Plasmodium* parasite culture.

The *P. knowlesi* strain A1-H.1 was cultured, as previously described.^41^ Parasites were grown in RPMI-1640 (Sigma-Aldrich, St. Louis, MO), supplemented with 2 g/L dextrose anhydrous, 0.05 g/L hypoxanthine, 0.5% AlbuMAX II (Invitrogen, Waltham, MA), and 10% (v/v) horse serum (Invitrogen). Human red blood cells (RBCs) were obtained from Duffy antigen-positive individuals from PlasmaLab (Everett, WA), and washed RBCs were added to 2% hematocrit in the culture media. The culture was maintained at 37°C with a hypoxic gas mixture of 90% N_2_, 5% CO_2_, and 5% O_2_. For time-course studies, high parasite density cultures were synchronized in three replicates twice using centrifugation with Nycodenz AG^®^ (Axis-shield Diagnostics Ltd, Dundee, Scotland) gradient solution 4 hours apart, retaining the upper brown portion in the first centrifugation and the lower RBC portion for the second centrifugation, to achieve the highest synchronization rate. After synchronization, synchrony was confirmed, and parasite density in the culture was determined by staining thin-film smear using a Hemacolor^®^ staining kit (HARLECO, Sigma Aldrich, St. Louis, MO) and counting parasites in a minimum of 2,000 RBCs using a microscope. Following the second synchronization, the culture was incubated and harvested at different time points. For kinetics of *Pk*LDH, infected RBC (iRBC) cultures that were incubated in the culture tubes in three replicates were harvested at 0, 1, 2, 6, 19, 21, 25, 27, 28, 29, and 30 hours. After centrifugation, the cell culture supernatant was collected, and the remaining iRBC pellets were washed with phosphate-buffered saline (PBS) and resuspended in PBS at the original culture volume. The total number of iRBCs was counted using an Accuri™ C6‐UV flow cytometer (BD Biosciences, San Jose, CA)

*Plasmodium cynomolgi* parasites were cultured in complete RPMI media supplemented with GlutaMAX^TM^ (Gibco, Waltham, MA) and 200 µM hypoxanthine (Calbiochem, San Diego, CA) as described previously.^40^ Rhesus monkey blood (BioIVT, Westbury, NY) was added to 5% hematocrit, and parasites in a plate were cultured at 37°C in a hypoxic gas mixture of 90% N_2_, 5% CO_2_, and 5% O_2_. Three lots of in vitro culture as both iRBC pellet and culture supernatant were prepared on the second day after synchronization and frozen prior to shipment to PATH (Seattle, WA) for the antigen quantification.

### Production of *Plasmodium* species-specific pLDH proteins.

PATH collaborated with the University of Queensland Protein Expression Facility (University of Queensland, St. Lucia, Australia) to develop *Plasmodium* species-specific pLDH proteins and human LDH protein for use as reference proteins. Based on the DNA sequence information available from GenBank, the full-length *ldh* genes specific for *P. falciparum*, *P. vivax*, *P. ovale curtisi*, and *P. malariae* and the human full-length *ldh* gene were synthesized and cloned into the prokaryotic expression vector pOPIN-NHis3C to express in Rosetta^TM^ (DE3)pLysS competent cells (Novagen, Madison, WI). Bacterial culture was performed in 1 L TB media (Novagen) containing antibiotics at 30°C for around 24 hours. Culture was harvested by centrifugation at 5,000 × *g* for 30 minutes at 4°C and resuspended in lysis buffer. After lysing using sonication, the lysate was centrifuged at 20,000 × *g* for 30 minutes at 4°C, and the supernatant representing the soluble fraction was isolated. The soluble fraction was subjected to an immobilized metal affinity chromatography using a HisTrap^TM^ Fast Flow column (GE Healthcare, Chicago, IL) followed by size-exclusion chromatography (SEC)^4^ using a HiLoad^®^ 26/600 Superdex^®^ 200 column (GE Healthcare, Chicago, IL). The SEC elution fractions were analyzed by resolving on 12% SDS-PAGE. The SEC elution fractions of interest were pooled and concentrated using an Amicon^®^ centrifuge filter (Sigma-Aldrich, St. Louis, MO). The sample was filtered through a 0.22 µm filter and stored at −80°C. The amount and purity of His-tag-containing recombinant pLDH and human LDH proteins were estimated by absorbance at 280 nm using NanoDrop^TM^ (Thermo Fisher Scientific, Waltham, MA) spectrophotometer and SDS-PAGE, respectively. All recombinant pLDH and human LDH proteins showed more than 95% purity (Supplemental Figure 1) and were kept at −80°C for long-term storage until future use.

### Quantification of malaria antigens.

The presence and quantification of *Pf*LDH, *Pv*LDH, and PanLDH in iRBC pellets, culture supernatant, clinical samples, and samples spiked with recombinant pLDH proteins were tested using the Q-Plex^TM^ Human Malaria Array (Quansys Biosciences, Logan, UT; hereafter the 5-Plex) to target five different analytes—HRP2, *Pf*LDH, *Pv*LDH, PanLDH, and C-reactive protein (CRP)—according to the manufacturer’s instructions.^39^ These analytes are identified using pairs of specific antibodies in which capture antibodies are printed as defined spots in a geometric planar array on the surface of each well and a mix of detection antibodies are used to identify the analytes captured on the defined spots. For the pLDH assays, capture antibodies shown to be specific for *P. vivax* and *P. falciparum* parasites were individually spotted for the capture of *Pv*LDH and *Pf*LDH, respectively, with a third spot representing a capture antibody to *Plasmodium* LDH as a PanLDH determinant. A broadly reactive PanLDH antibody was used as the detector antibody for all three assays (*Pf*LDH, *Pv*LDH, and PanLDH). For comparison of reactivity pattern, iRBC pellets of synchronized *P. cynomolgi* and *P. knowlesi* cultures and *P. vivax* clinical samples were diluted at 200 and 40 p/µL of parasite density and tested on the 5-Plex. For the samples at high concentration, which falls outside the standard range, the samples were subsequently diluted 1:20 and 1:400 with assay diluent, and the dilution factors were applied for the final concentration of antigens.

### Statistical analysis.

All data were analyzed using GraphPad Prism (version 5.0; La Jolla, CA). Pearson’s correlation coefficient was calculated for the PanLDH level based on the 5-Plex and for parasite density based on microscopy or qPCR. The results relative to ratios of PanLDH*/Pf*LDH and PanLDH/*Pv*LDH are presented as mean ± SD. Student’s *t*-test was used to determine significant differences between ratio values of PanLDH and species-specific pLDH of recombinant and native proteins, estimated by reference assay. In addition, a two-way analysis of variance (ANOVA) with Sidak multiple comparison test was used to establish statistical significance between groups (*P* < 0.05).

## RESULTS

### Performance of the 5-Plex for reactivity to recombinant and native pLDH proteins for *P. falciparum*, *P. vivax*, *P. malariae,* and *P. ovale.*

**Figure 1.**
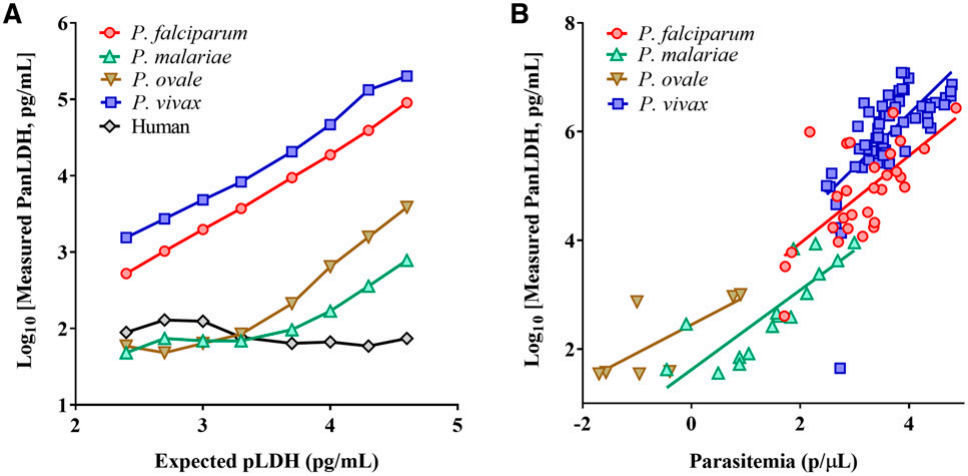
Binding reactivity of recombinant or native pLDH proteins to PanLDH spot. (**A**) Expected concentration vs. measured concentration of recombinant pLDH and human LDH proteins. Depicted are line charts including eight data points generated with 2-fold serial dilutions of pLDH and human LDH proteins. (**B**) Scatter plots and best fit lines between parasite density and PanLDH level in clinical blood samples estimated by microscopy or qPCR, and the 5-Plex, respectively. Any samples with *P. malariae*, *P. ovale,* or *P. vivax* infections, which had no available data for parasite density, were excluded from analysis. Pearson correlation coefficient was calculated using GraphPad Prism. This figure appears in color at www.ajtmh.org.

Dilution series of whole blood samples spiked with recombinant pLDH proteins specific for *P. falciparum*, *P. vivax*, *P. malariae,* and *P. ovale curtisi*, and clinical whole blood samples infected with each of four *Plasmodium* species were tested on the 5-Plex to examine the presence and level of reactivity to the assays for PanLDH, *Pf*LDH, and *Pv*LDH. Analysis showed that good specificity on *Pf*LDH and *Pv*LDH spots was found with recombinant *Pf*LDH and *Pv*LDH proteins, respectively (Supplemental Figure 2A). Interestingly, recombinant *P. ovale-*specific pLDH (*Po*LDH) and *P. malariae-*specific pLDH (*Pm*LDH) proteins showed reactivity with both PanLDH and *Pf*LDH spots of the 5-Plex. However, a notable difference was observed with the relative signal from the PanLDH spot, in which both *Po*LDH and *Pm*LDH recombinant proteins showed relatively less signal, resulting in lower measured concentration as compared with recombinant *Pf*LDH or *Pv*LDH proteins for a given concentration (Figure 1A).

For the reactivity to native pLDH from four *Plasmodium* species, the reactivity with PanLDH, *Pf*LDH, and *Pv*LDH spots of the 5-Plex was examined. Native *Po*LDH and *Pm*LDH in clinical samples behaved similarly to their recombinant counterparts with both the PanLDH and *Pf*LDH spots on the 5-Plex (Supplemental Figure 2B). Furthermore, the relation between level of PanLDH and parasite density was assessed with clinical samples infected by each *Plasmodium* species (Figure 1B). The correlation analysis demonstrated that PanLDH level showed significant positive correlations with parasite density determined by either microscopy or qPCR for *P. falciparum* (*N* = 29, r = 0.670, *P* < 0.0001), *P. vivax* (*N* = 60, r = 0.674, *P* < 0.0001) and *P. malariae* (*N* = 16, r = 0.834, *P* < 0.0001) but relatively poor correlation with parasite density for *P. ovale* (*N* = 7, r = 0.737, *P* = 0.059).

**Figure 2.**
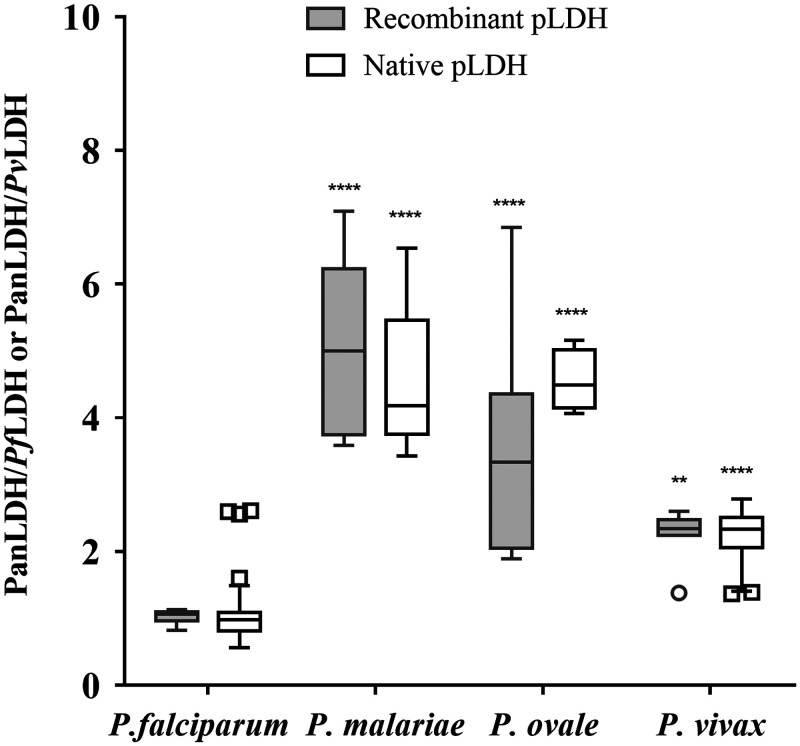
Reactivity of recombinant and native pLDH proteins. Any samples showing antigen-negative results by the 5-Plex were excluded from analysis. Depicted are box and whisker plots for ratios of PanLDH/*Pf*LDH for the *P. falciparum*, *P. malariae*, and *P. ovale* columns or ratio of PanLDH/*Pv*LDH for the *P. vivax* column for each source of pLDH (recombinant and native). The ends of the boxes correspond to the 25th and 75th percentiles, respectively, and the middle line corresponds to the median. Two-way ANOVA plus Sidak multiple comparison test was used to determine the statistical significance against ratio values of *P. falciparum*. Significant differences (**** *P* < 0.0001, ** *P* < 0.001).

The ratio of PanLDH to species-specific pLDH was similar for both native pLDH from clinical samples and the same recombinant proteins across the four *Plasmodium* species (Table 1, Figure 2). Specifically, the ratio was 1.03 ± 0.1 and 1.12 ± 0.55 for recombinant and native *Pf*LDH proteins, 4.25 ± 0.78 and 4.56 ± 1.0 for recombinant and native *Pm*LDH proteins, 3.10 ± 1.04 and 4.56 ± 0.46 for recombinant and native *Po*LDH proteins, and 2.26 ± 0.38 and 2.24 ± 0.37 for recombinant and native *Pv*LDH proteins (all *P* values statistically not significant). The PanLDH concentration measurement in the 5-Plex is calibrated to recombinant *Pf*LDH and so the ratio of close to 1 for PanLDH/*Pf*LDH is to be expected.

**Table 1:** Comparison of four *Plasmodium*-specific recombinant pLDH proteins against their corresponding native counterparts related to their binding reactivity

	Recombinant protein				Clinical sample			
Reactivity	*Pf*LDH (n = 8)	*Pm*LDH (n = 8)	*Po*LDH (n = 8)	*Pv*LDH (n = 8)	*P. falciparum* (n = 29)	*P. malariae* (n = 15)	*P. ovale* (n = 4)	*P. vivax* (n = 58)
	Pan/*Pf* ratio			Pan/*Pv* ratio	Pan/*Pf* ratio			Pan/*Pv* ratio
Range	0.82–1.13	3.59–5.49	1.89–4.38	1.38–2.6	0.56 − 2.61	3.43–6.54	4.07–5.16	1.37–2.79
Mean	1.03	4.25	3.10	2.26	1.12	4.56	4.56	2.24
SD	0.1	0.78	1.04	0.38	0.55	1.0	0.46	0.37
t-test	–	–	–	–	*P* = 0.63	*P* = 0.33	*P* = 0.27	*P* = 0.89

Pan/*Pf* = PanLDH/*Pf*LDH; Pan/*Pv* = PanLDH/*Pv*LDH. Descriptive statistics for the ratio of PanLDH concentration to *Pf*LDH or *Pv*LDH concentration as indicated, determined via the 5-Plex. Any clinical samples that showed unquantifiable or infection-negative test results by the 5-Plex were excluded from analysis. *P* values correspond to comparison of different antigen sources.

### Performance of reference assay evaluated with in vitro cultures of *P. cynomolgi* and *P. knowlesi.*

To examine the binding patterns of pLDH from *P. knowlesi* and *P. cynomolgi* on each assay for PanLDH, *Pf*LDH, and *Pv*LDH, a series of dilutions (200 and 40 p/µL) of whole blood sample spiked with synchronized *P. cynomolgi* and *P. knowlesi* iRBC pellets were tested with the 5-Plex. The results were comparable to that of a clinical *P. vivax* blood sample. The results showed the similar reactivity between *Pk*LDH and *Pv*LDH proteins for binding to the PanLDH and *Pv*LDH spots, and with comparable PanLDH/*Pv*LDH ratio values between these two *Plasmodium* species at 2.7 for *Pk*LDH and 2.8 for *Pv*LDH at 200 p/µL of parasite density (Table 2). However, we found that *Pc*LDH of *P. cynomolgi* only reacted with the *Pv*LDH spot and not to the PanLDH spot.

**Table 2:** Concentration estimates for PanLDH and *Pv*LDH determined from cell cultures of *P. cynomolgi* and *P. knowlesi* and a clinical sample of *P. vivax*

	*P. cynomolgi*			*P. knowlesi*			*P. vivax*		
Parasitemia (p/mL)	PanLDH (pg/mL)	*Pv*LDH (pg/mL)	Pan/*Pv* ratio	PanLDH (pg/mL)	*Pv*LDH (pg/mL)	Pan/*Pv* ratio	PanLDH (pg/mL)	*Pv*LDH (pg/mL)	Pan/*Pv* ratio
200	28.8*	4,603.2	0.01	890.3	332.1	2.7	30,758.1	11,183.0	2.8
40	29.6*	1,102.5	0.03	207.2	78.6	2.6	8,064.1	2,805.8	2.9

Pan/*Pv* = PanLDH/*Pv*LDH.

* For the Pan/*Pv* calculation, the PanLDH value from background binding was used.

### Time-course study on *Pk*LDH expression over parasite stages of *P. knowlesi.*

**Figure 3.**
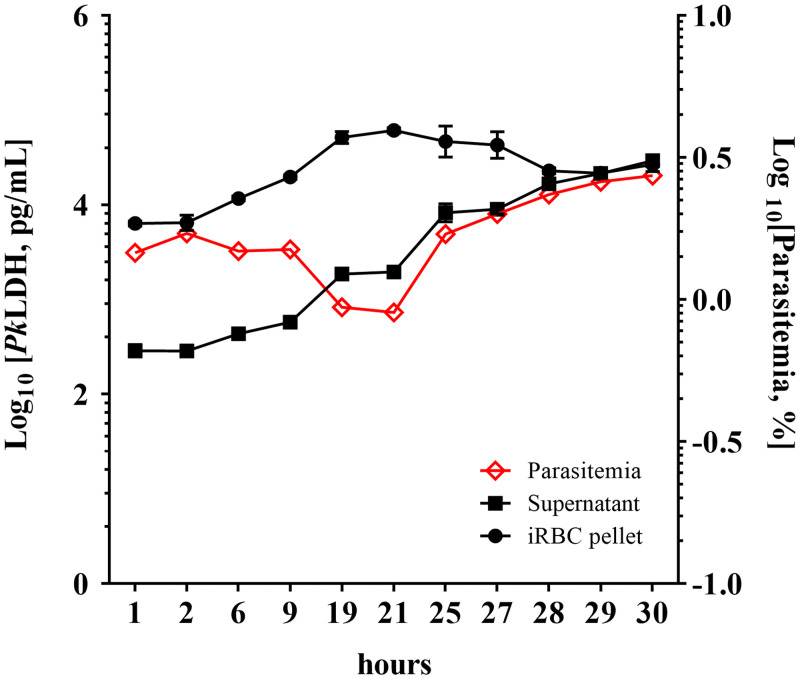
*Pk*LDH accumulation in *P. knowlesi* cultures. Percentage parasitemia in synchronized cultures was monitored using microscopy, and the *Pk*LDH level in the iRBC pellet and supernatant from 1 mL of synchronized culture was quantified using *Pv*LDH assay and in three replicates over 30 hours. For estimating parasitemia, one of three replicates was examined. Time zero represents time of synchronization. Error bars indicate standard deviation of the mean. This figure appears in color at www.ajtmh.org.

For the time-course study, *Pk*LDH protein in the iRBC pellet and supernatant obtained from in vitro culture over 30 hours after synchronization were tested by the 5-Plex. Our results (Figure 3) demonstrated a life cycle for *P. knowlesi* of approximately 28 hours based on the time gap between the first ring stage and the second ring stage (Supplemental Table 1), similar to the previously published finding of 27 hours.^39^ Although most of the *Pk*LDH was found in iRBC pellets rather than in the supernatant, both pellets and supernatant showed progressively increased concentration of *Pk*LDH (∼8 times more after 19 hours compared with 1 hour for both pellets and supernatant) during ring and trophozoite stages (Figure 3, Supplemental Table 1). The maximal amount of *Pk*LDH is reached in the pellet during the schizont stage (21 to 27 hours), but a continuous accumulation of *Pk*LDH released from the schizont rupture in the supernatant was observed over this period of time. Interestingly, during the first life cycle, parasite density seems to be decreased during the schizont stage, and the number of parasites in the second life cycle is increased after the released cells infect the fresh cells.

## DISCUSSION

The human malaria array (5-Plex) was developed to provide quantitative measurement of the malaria antigens HRP2, *Pf*LDH, and *Pv*LDH and a qualitative measure for all malaria species (PanLDH) as well as measure the human biomarker CRP.^39^ The pLDH spots used in this reference assay were screened for specificity against *Pf*LDH and P*v*LDH^38^^,^^39^ as well as characterized for their utility to screen for *hrp2/hrp3* deletions both in whole blood pellets^39^ and dried blood spots.^42^ We demonstrated that the malaria array is a valuable tool in the detection and diagnosis of *P. falciparum* and *P. vivax*, using the described biomarkers.^39^^,^^42^ However, its capability to diagnose less-common *Plasmodium* species such as *P. malariae* and *P. ovale*, and two simian *Plasmodium* species, *P. knowlesi *and* P. cynomolgi*, which have been recognized to cause human infection, has not been fully explored. As the 5-Plex includes a PanLDH spot detecting all *Plasmodium* species, in this study, we first evaluated the potential cross-reactivity of pLDH proteins from these parasites against pan-malaria and species-specific spots using both recombinant and native proteins.

The recombinant pLDH proteins of *P. falciparum*, *P. malariae*, *P. ovale curtisi*, and *P. vivax* offer great diagnostic value for the appropriate evaluation of the assay performance when there are limited clinical blood samples. Our study demonstrated that these recombinant pLDH proteins behave similarly to native pLDH proteins from corresponding *Plasmodium* species as demonstrated by PanLDH/species-specific pLDH ratio while being distinct from the equivalent host LDH protein (Table 1). However, there seemed to be different binding characteristics resulting in different signal responses from the PanLDH spot on the 5-Plex: the highest signal was observed with recombinant *Pv*LDH followed by recombinant *Pf*LDH, recombinant *Po*LDH, and then recombinant *Pm*LDH (Supplemental Figure 2A).

The significant variation of PanLDH values in the estimated concentration of recombinant pLDH proteins suggest two possibilities: 1) some minor variance in the amino acid sequence of the conserved region or that of the surrounding area that enhances or reduces the antigen–antibody reaction, and 2) inefficient protein folding during protein expression/purification might have occurred when using a prokaryotic expression system. The second possibility may be addressed by evaluating the native pLDH proteins purified from different *Plasmodium* species, but obtaining native proteins from live parasites is a major challenge, especially with species of *P. vivax*, *P. ovale*, and *P. malariae*, for which continuous culture systems have not been established unlike *P. falciparum* strains.^43^^–^^45^ Alternatively, nonhuman primate models could offer the strategies of producing non-*P. falciparum* parasites and their respective native proteins.^46^

The pLDH amount measured as PanLDH correlates to parasite density equally across species, as shown by the normalized binding reactivity of native pLDH proteins with respect to the given parasite number (Supplemental Figure 3). The results suggest that this could be an artifact derived from the protein purification processes.

Additionally, both *Pm*LDH and *Po*LDH were found to react not only to the PanLDH spot but also to the *Pf*LDH spot, suggesting an overlap in antigenic epitopes of pLDH possessed by *P. falciparum*, *P. malariae*, and *P. ovale*. In our preliminary study, cross-reactivity between *P. malariae* and the *Pf*LDH band on a few commercial RDTs was also observed (data not shown). It will therefore be of interest to investigate the extent of the level of cross-reactivity, which may have a significant influence on interpreting diagnostic test results. The PanLDH/species-specific pLDH ratio values for *P. malariae, P. ovale*, and *P. vivax* were significantly different from that for *P. falciparum* (Figure 1), suggesting the potential to use this ratio difference in differentiating *Plasmodium* species on the 5-Plex.

The reactivity patterns of pLDH from *P. vivax* and *P. cynomolgi* were compared with *P. knowlesi*, which is more distantly related to *P. vivax*. *P. cynomolgi* causes periodic relapses by dormant hypnozoites, early infectious gametocyte formation, and invasion of Duffy blood group-positive reticulocytes, and thus offers a robust model for *P. vivax* infection in the rhesus monkey model.^40^^,^^47^^,^^48^ This study revealed that *P. cynomolgi* failed to exhibit cross-reactivity to the PanLDH spot of the 5-Plex but did show reactivity to the *Pv*LDH spot (Table 2). Unfortunately, there is no information available on the specific epitopes for PanLDH-specific capture and detector antibodies used in the 5-Plex. We aligned the amino acid sequences of the pLDH proteins from four different *Plasmodium* species (obtained from the GeneBank database) and found a conserved region (the region forming the loop structure) as the potential site recognized by pan-malaria antibodies and several species-specific regions as described previously (Supplemental Figure 4).^49^^–^^51^ We did not find any obvious sequence variance in the potential pan-epitope region of *Pc*LDH protein as compared with three other parasite species including *P. falciparum, P. vivax,* and *P. knowlesi* that would explain our observation. It would be interesting to explore binding characteristics of commercially available pan-malaria antibodies to the laboratory-adopted *P. cynomolgi* strain or recombinant *Pc*LDH reagents and investigate the extent of sequence conservation within the conserved region of *Pc*LDH protein from the cultured *P. cynomolgi* in a future study.

*Pk*LDH exhibited significant cross-reactivity to both the PanLDH and *Pv*LDH spots, but no or little cross-reactivity with the *Pf*LDH spot on the 5-Plex. A previous study looking at cross-reactivity between pLDH proteins from different *Plasmodium* species and different antibodies to pLDH highlighted that *Pk*LDH can react to antibodies specific to both *Pf*LDH and *Pv*LDH.^33^ In the 5-Plex, only cross-reactivity between *Pk*LDH and the *Pv*LDH antibody pair was observed. Additionally, the PanLDH/*Pv*LDH ratio for *P. knowlesi* was similar to that seen when measuring *Pv*LDH from the *P. vivax* clinical samples. Although the PanLDH and *Pv*LDH spots on the 5-Plex can detect the presence of the *Pk*LDH antigen, they were unable to distinguish *P. knowlesi* from *P. vivax* infection. To differentiate *P. knowlesi* from other *Plasmodium* species, development of an expanded array that hosts a *P. knowlesi-*specific antigen assay is required.

This is the first study to demonstrate the dynamic kinetic expression of *Pk*LDH in a culture system. The results exhibited accumulation of *Pk*LDH over time in culture, except with a slight decrease in *Pk*LDH concentration seen in samples from the late schizont stage at around 25–28 hours. The pellet fraction contains the largest total amount of *Pk*LDH until 29 hours after synchronization. At an inflection point where more *Pk*LDH is contained in the supernatant, the erythrocytic cycle is completed through the rupturing of the schizonts to create new ring-stage parasites. The overall decrease in *Pk*LDH seen starting at hour 25 may be explained by the lability of *Pk*LDH and consequently an overall drop in *Pk*LDH concentration until the parasites mature again. It should also be noted that the reactivity pattern of *Pk*LDH proteins as a function of PanLDH/*Pv*LDH ratio was maintained at 2.11 ± 0.24 and 2.35 ± 0.24 in both cell pellet and supernatant compartments, respectively, throughout all development stages (Supplemental Table 2).

The differential binding of pLDH from the different *Plasmodium* species investigated and observed in this study across the 5-Plex *Pf*LDH, *Pv*LDH, and PanLDH assays echoes results from previous studies investigating both antibody reactivity to pLDH and RDT reactivity to pLDH.^26^^,^^33^^,^^52^^,^^53^ The results presented here are most likely specific to the particular anti-pLDH antibodies used in this 5-Plex assay. In a similar manner, the pLDH assay components of widely used RDTs should be evaluated for cross-reactivity to the different human malaria species, especially to *P. ovale*, *P. malariae*, and *P. knowlesi*, and also to the less-common species known to infect humans, including *P. cynomolgi* and *P. simium*. This will be particularly relevant to countries approaching elimination and/or that have a significant relative burden of *P. knowlesi*.^54^^,^^55^ Between the emergence of *hrp2/hrp3* deletions in *P. falciparum* infections and pLDH as the most commonly used target analyte for non-*P. falciparum* malaria RDTs, understanding the dynamics and cross-reactivity of pLDH between the different human *Plasmodium* species is essential. Additionally, given the known cross-reactivities between different *Plasmodium* species, new diagnostic markers for better differentiating *Plasmodium* species should be explored.

## Supplemental tables


Supplemental materials
