# Supplementary material for: Diagnostic Characteristics of Lactate Dehydrogenase on a Multiplex Assay for Malaria Detection Including the Zoonotic Parasite Plasmodium knowlesi
Source: Am J Trop Med Hyg. 2021 Nov 15;106(1):275–82. doi: 10.4269/ajtmh.21-0532 (PMC8733487; doi:10.4269/ajtmh.21-0532)
Supplement: Supplementary file 1 [file tpmd210532.SD1.pdf]

Supplemental materials

Supplemental Figure 1. SDS-polyacrylamide gel electrophoresis (PAGE) analysis of recombinant pLDH and human LDH proteins. To evaluate the protein purity, recombinant protein in processing and/or final sample was run on SDS-PAGE. The gel was stained with Coomassie blue G250. Migration of pre-stained molecular weight (MW) standard of proteins is shown in the first lane. Protein purity (expressed as a percentage) was estimated by band intensity analysis using Image Lab software (Bio-rad, Hercules, CA). A) *Pf*LDH, 97%; B) *Pm*LDH, 99%; C) *Po*LDH, > 99%; D) *Pv*LDH, 97%; and E) human LDH, 99%.

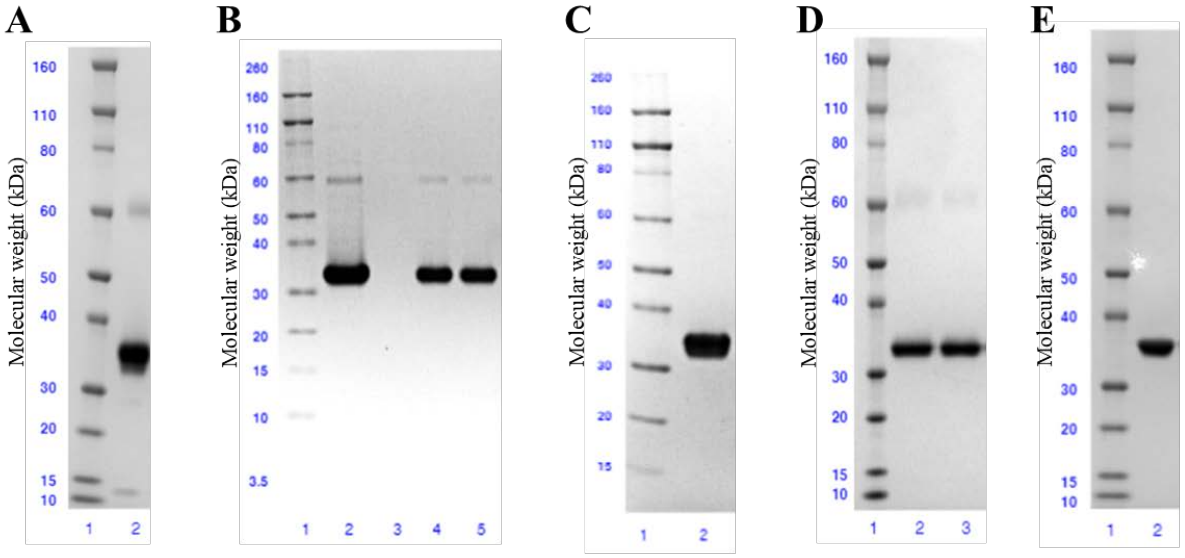

A)
1. MW standard
2. Final (filtered)

B)
1. MW standard
2. Pooled fractions
3. Concentrator waste
4. After concentration
5. Final (filtered)

C)
1. MW standard
2. Final (filtered)

D)
1. MW standard
2. Pooled
3. Final (filtered)

E)
1. MW standard
2. Final (filtered)

Supplemental Figure 2. The reactivity of recombinant and native pLDH proteins on PanLDH, *Pf*LDH, and *Pv*LDH spots of the reference assay. A) A serial dilution with recombinant pLDH proteins for four *Plasmodium* species including *P. falciparum*, *P. vivax*, *P. malariae*, and *P. ovale curtisi*, and recombinant human LDH protein as a control was evaluated with the 5-Plex, detecting the range between LDH concentrations of 250–40,000 pg/mL. B) Clinical blood samples with different parasite infection (*P. falciparum* [n =29], *P. vivax* [n = 60], *P. malariae* [n =15], and *P. ovale* [n =4] were tested on the 5-Plex. Parasitemia in samples was determined using microscopy or PCR methods. Plots show the log of the concentration of expected recombinant pLDH protein or parasitemia (x-axis) versus the log of the signal intensity from PanLDH, *Pf*LDH, and *Pv*LDH spots measured with neat samples (y-axis). The lines in (B) represent regression lines of best fit for each set of clinical samples.

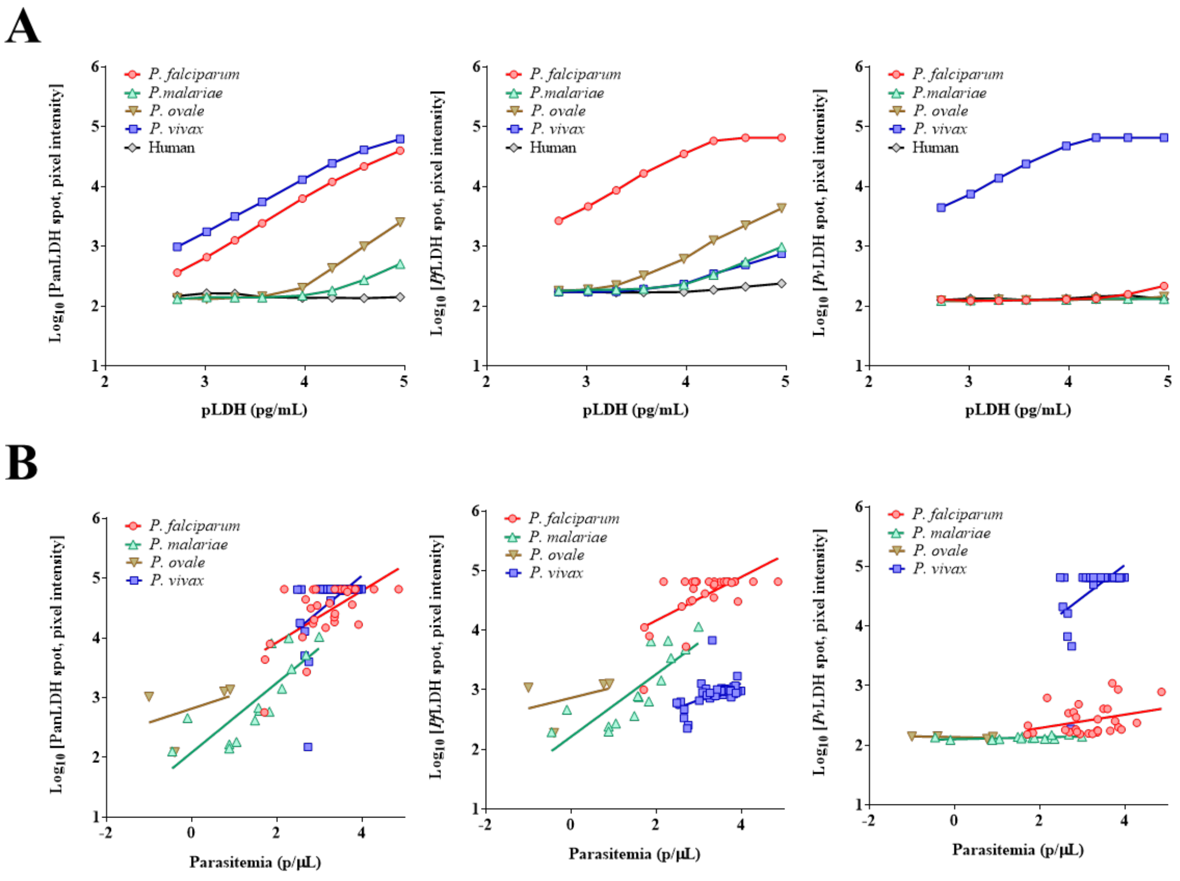

Supplemental Table 1. Percentages of *P. knowlesi*–infected red blood cells at different stages over 30 hours of culture after synchronization.

Hour post synchronization	Parasitemia (%)	Intraerythrocytic stages		
		% ring	% trophozoite	% schizont
1	1.49	100	0	0
2	1.46	100	0	0
6	1.71	100	0	0
9	1.48	47	50	3
19	1.5	3	94	3
21	0.94	0	9	91
25	0.9	0	0	100
27	1.7	30	0	70
28	2.0	89	0	11
29	2.34	93	0	7
30	2.6	98	2	0

Supplemental Figure 3. Native pLDH amount per parasite estimated by the 5-Plex. The measured PanLDH values were normalized against the parasite density examined by microscopy or qPCR. Any clinical samples which showed unquantifiable or infection-negative test results by the 5-Plex were excluded from analysis. Depicted are box plots of PanLDH amount produced by a parasite of *Plasmodium* species as indicated. The medians are shown by the horizontal lines inside the boxes, the 25th and 75th percentiles are shown as the bottoms and tops of the boxes, and data points outside the range represent outliers. One-way ANOVA plus Sidak multiple comparison test was used to determine the statistical significance relative to PanLDH values measured with *P. falciparum* samples. Significant differences (** $p < 0.001$). fg, femtogram; ns, not significant

Log_{10} [PanLDH, fg/parasite]

Supplemental Figure 4. Alignment of pLDH sequences from various *Plasmodium* species. Clustal Omega multiple sequence alignment program (http://www.clustal.org/omega/) was used to align multiple pLDH amino acid sequences from strains of *Plasmodium* species: *P. cynomolgi* Strain B (PCYB), *P. cynomolgi* Strain M (PCYM), *P. knowlesi* strain H (PKNH), *P. knowlesi* Malayan (PKNOH), *P. vivax* P01 (PVP01), *P. vivax* Sal-1 (PVX), *P. falciparum* 3D7 (PF3D7), *P. falciparum* Dd2 (PFDD2), *P. ovale* GH01 (POCGH01), and *P. malariae* UG01 (PMUG01). A conserved region and several species-specific regions are indicated.[1, 2, 3] The figure was created using MView (https://www.ebi.ac.uk/Tools/msa/mview/). Coverage and percentage identity values are indicated as cov and pid, respectively.

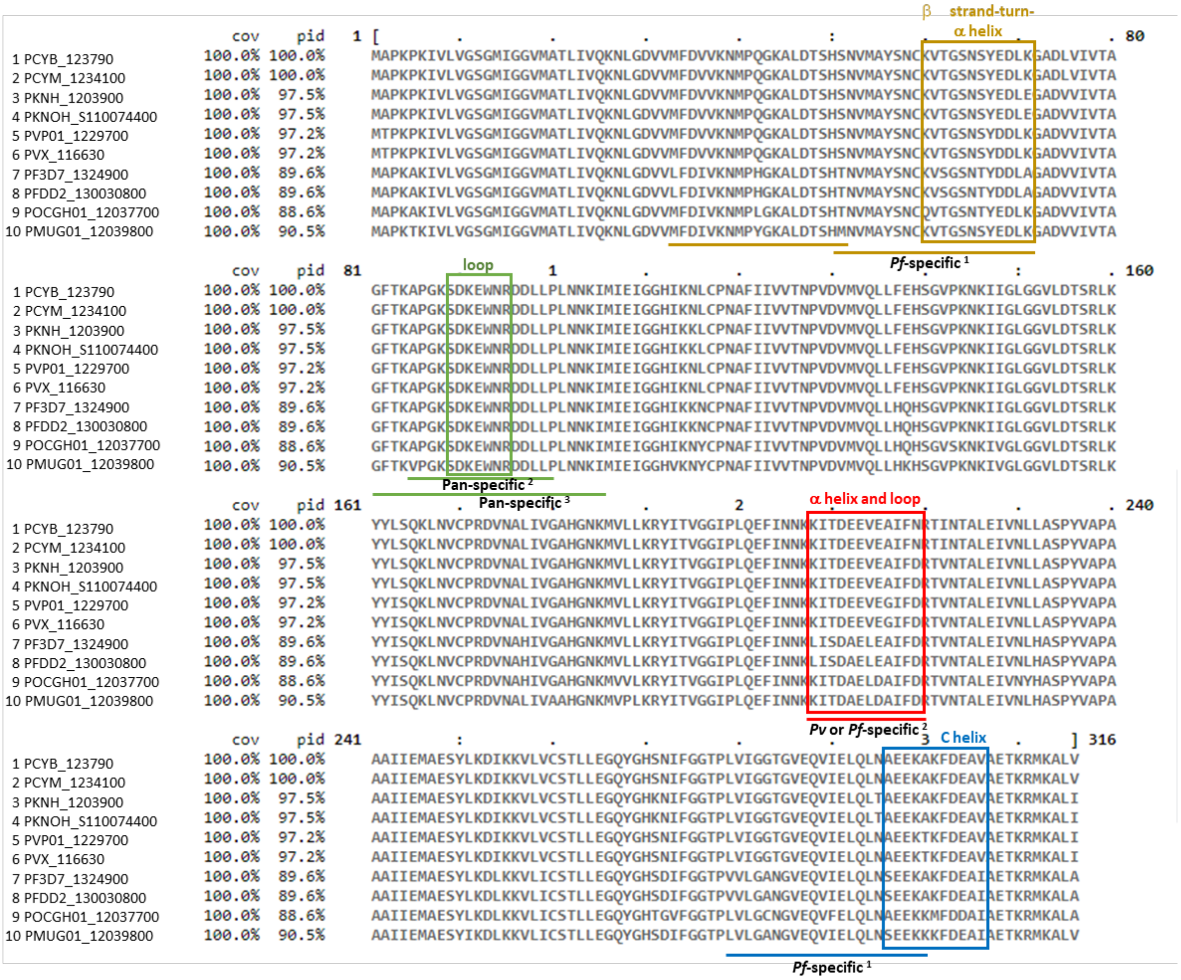

Supplemental Table 2. PanLDH/*Pv*LDH ratio measured with the RBC pellet and supernatant materials over the erythrocytic cycle of the *P. knowlesi* culture.

	**RBC pellet**			**Supernatant**		
**Hour post synchronization**	**PanLDH (pg/mL)**	***Pv*LDH (pg/mL)**	**Pan/*Pv***	**PanLDH (pg/mL)**	***Pv*LDH (pg/mL)**	**Pan/*Pv***
1	73,974.3	31,775.3	2.33	823.1	296.1	2.78
2	75,550.5	32,739.1	2.31	638.7	293.4	2.18
6	135,437.4	58,408.3	2.32	1,001.6	447.5	2.24
9	224,167.8	98,373.0	2.28	1,378.5	589.3	2.34
19	461,451.6	258,914.0	1.78	5,018.9	1,905.4	2.63
21	523,228.5	305,626.5	1.71	5,096.4	2,001.0	2.55
25	439,765.7	244,306.3	1.80	18,473.4	8,559.6	2.16
27	454,845.4	221,902.3	2.05	22,611.3	9,234.2	2.45
28	260,037.4	114,659.1	2.27	34,634.2	16,968.0	2.04
29	242,654.4	107,746.1	2.25	46,014.4	21,959.7	2.10
30	301,172.8	135,057.2	2.23	73,191.8	29,993.0	2.44
Mean			2.11			2.35
SD			0.24			0.24

SD: standard deviation, CV: coefficient of variation, Pan/*Pv*: Pan LDH/*Pv*LDH

References

1. Verma P, Biswas S, Mohan T, Ali S, Rao DN, 2013. Detection of histidine rich protein & lactate dehydrogenase of *Plasmodium falciparum* in malaria patients by sandwich ELISA using in-house reagents. *Indian J Med Res* 138: 977–987.

2. Hurdayal R, Achilonu I, Choveaux D, Coetzer THT, Goldring JPD, 2010. Anti-peptide antibodies differentiate between plasmodial lactate dehydrogenases. *Peptide* 31: 523–532.

3. Tomar D, Biswas S, Tripathi V, Rao DN, 2006. Development of diagnostic reagents: raising antibodies against synthetic peptides of PfHRP-2 and LDH using microsphere delivery. *Immunobiol* 211: 797–805.
